# The Impact of Vitamin D Deficiency on Outcomes After Surgery for Adolescent Idiopathic Scoliosis: A Systematic Review

**DOI:** 10.7759/cureus.96282

**Published:** 2025-11-07

**Authors:** Mariette Anto, Israa Elkashif, Sindhu Vithayathil, Maryam Walizada, Gowtham Siddi, Srirachana Reddy Gumireddy, Ann Kashmer Yu

**Affiliations:** 1 General Medicine, King's Mill Hospital, Sutton-in-Ashfield, GBR; 2 Psychiatry, Royal College of Surgeons in Ireland, Dublin, IRL; 3 General Medicine, American University of Antigua, Coolidge, ATG; 4 Internal Medicine, Mary Washington Hospital, Fredericksburg, USA; 5 Medicine, NRI Academy of Medical Sciences, Guntur, IND; 6 Internal Medicine, Apollo Institute of Medical Sciences and Research, Hyderabad, IND; 7 Internal Medicine, California Institute of Behavioral Neurosciences and Psychology, Fairfield, USA

**Keywords:** adolescent idiopathic scoliosis (ais), scoliosis surgery, spine surgury, vitamin-d, vitamin d deficiency

## Abstract

Adolescent idiopathic scoliosis is a common spinal orthopedic condition that is associated with a high prevalence of vitamin D deficiency. Vitamin D is known to affect bone health, and we conducted a systematic review to assess the impact of vitamin D deficiency on patients diagnosed with adolescent idiopathic scoliosis who undergo surgery. We followed the Preferred Reporting Items for Systematic Reviews and Meta-Analyses (PRISMA) 2020 checklist for our systematic review, utilizing various databases to conduct a thorough analysis of the current literature. We included specific search terms related to vitamin D deficiency and adolescent idiopathic scoliosis, and considered articles published between 2010 and 2025, focusing on papers with free full-text access. We used the PRISMA flowchart and initially identified 1,286 articles using the keywords for our search strategy across the databases. After removing duplicates, conducting title screening, assessing abstracts, and evaluating quality, we included 12 research papers in this review. Our review focused on the prevalence of vitamin D deficiency in adolescent idiopathic scoliosis patients and the impact of vitamin D deficiency on patients undergoing surgical management. This study was inconclusive in proving how significant the impact of vitamin D deficiency was in surgical adolescent idiopathic scoliosis patients, and we recommended conducting additional research to establish the importance of correcting vitamin D deficiency pre-operatively. However, considering that vitamin D has been linked to poorer surgical outcomes in most of the studies included in our review, we recommend routine screening for vitamin D in adolescent idiopathic scoliosis patients and its correction pre-operatively.

## Introduction and background

Adolescent idiopathic scoliosis (AIS) is a common orthopedic condition that affects adolescents, resulting in a three-dimensional spinal malformation that causes lateral shift and vertebral rotation of the spine [1-4]. The prevalence of scoliosis is 1.5-3 times higher in girls than in boys [5,6]. Although the exact cause of AIS is unknown, there are different speculations, including genetic, metabolic, neuromuscular, and environmental factors [7]. Bone mineral density is one of the factors that disrupts the mechanical stability of the bone [8]. A spinal curvature greater than 10° of the Cobb angle on a plain anteroposterior X-ray is the main diagnostic feature [9]. Surgical intervention is considered if the Cobb angle exceeds 40° [8-10]. Mild cases are treated with physical therapy and rehabilitation, and moderate cases usually require bracing to correct the spinal deformity [9].

Vitamin D is crucial for musculoskeletal development and function [2]. It facilitates intestinal calcium absorption, maintains calcium-phosphate balance, and regulates osteoblast proliferation [4]. High vitamin D levels have been associated with increased bone mineral density (BMD), a reduced risk of osteoporotic fractures, and improved neuromuscular function [2,11]. There is some evidence that low vitamin D levels are associated with osteopenia and poor bone density, which have been observed in patients with AIS [2,12,13]. This has been observed in both the axial and peripheral skeleton of AIS patients [2]. AIS patients had a higher prevalence of vitamin D deficiency (VDD) than the overall population [4,14], and it is thought to be linked to the onset and progression of AIS [4,14]. According to one study, 41.43% of individuals with AIS had VDD, whereas 36.19% had vitamin D insufficiency [15]. It has also been thought that treating the VDD may significantly improve bone health and possibly lower the risk of curve progression in these patients [16].

Therefore, some studies have concluded that vitamin D can predict an increase in Cobb angle [5,8], worse patient outcomes [17], poor post-operative outcomes [4,16], and increased back pain [16]. VDD is also associated with the etiopathogenesis of AIS [2]. On the other hand, other studies have concluded that vitamin D correction does not improve outcomes [18], and AIS patients do not need to be screened for low vitamin D levels [19].

Although previous systematic reviews and meta-analyses have been published to determine the incidence of VDD in AIS patients, there are no reviews examining the effect of low vitamin D levels on AIS patients undergoing surgical management [14,19]. Therefore, we conducted a systematic review to examine the current literature and determine how VDD affects AIS and surgical outcomes, specifically the prevalence of VDD in AIS patients, the correlation between low vitamin D levels and back pain, Cobb angle, curve progression, and therapeutic implications.

## Review

Materials and methods

The methodology of this systematic review is outlined according to the Preferred Reporting Items for Systematic Reviews and Meta-Analyses (PRISMA) guidelines [20].

Search Strategy

We systematically reviewed articles from the following databases: PubMed, PMC, Medline, Cochrane, MDPI, and ScienceDirect to identify suitable articles (papers) that can be included in the study. We used a combination of search terms, including vitamin D, scoliosis, adolescent idiopathic scoliosis, surgery, using Boolean operators (and, or) and PubMed MeSH. Table 1 below shows the databases used, search strategy used, number of papers identified, and date of search.

**Table 1:** Search strategy, database, number of papers identified, and date of search.

Search strategy	Database used	No. of papers identified	Date searched
(Vitamin D OR colecalciferol OR Vitamin D deficiency OR ("Vitamin D/adverse effects"[Majr] OR "Vitamin D/metabolism"[Majr] OR "Vitamin D/pharmacokinetics"[Majr] OR "Vitamin D/pharmacology"[Majr] OR "Vitamin D/physiology"[Majr])) AND (Adolescent Idiopathic Scoliosis OR scoliosis OR ("Scoliosis/diagnosis"[Majr] OR "Scoliosis/pathology"[Majr] OR "Scoliosis/physiopathology"[Majr] OR "Scoliosis/prevention and control"[Majr] OR "Scoliosis/rehabilitation"[Majr] OR "Scoliosis/surgery"[Majr] OR "Scoliosis/therapy"[Majr]) OR (spinal fusion OR ("Spinal Fusion/adverse effects"[Majr] OR "Spinal Fusion/instrumentation"[Majr] OR "Spinal Fusion/methods"[Majr])))	PubMed, Medline	173	May 3, 2025
Vitamin D AND adolescent idiopathic scoliosis	PMC	532	May 5, 2025
"vitamin D" AND "adolescent idiopathic scoliosis"	Cochrane	15	May 3, 2025
Vitamin D and adolescent idiopathic scoliosis	ScienceDirect	560	May 3, 2025
Vitamin D and scoliosis	MDPI	6	March 9, 2025
Total number of papers identified	-	1,286	-

Inclusion and Exclusion Criteria

Inclusion criteria: We included articles published between 2010 and 2023, written in English. Studies with human participants of all genders were included in our review. We only included studies with patients who were diagnosed with AIS. A few studies, which passed the screening process but whose full text was not freely available, were obtained through NHS institution access via OpenAthens or requested at the library at King's Mill Hospital, Sherwood Forest Hospitals NHS Foundation Trust.

Exclusion criteria: We were unable to obtain full-text articles for some publications, which were excluded accordingly. Articles that stated spinal fusion surgery but did not specifically mention patients diagnosed with AIS were excluded. Papers that did not meet the requirements when using the quality assessment tools were also excluded. The study was not limited by the type of publication or sample size, but grey literature, published abstracts, and proposal papers were excluded.

Selection/Screening Process

All identified articles from the various databases were transferred to Zotero. Duplicates were first removed. Two separate authors screened the papers independently by reviewing the titles and types of papers. The papers were then further shortlisted by reviewing the full text, and the relevant ones were selected. If consensus could not be reached between the two authors, those papers were discussed with other co-authors to reach a final decision. We then went through the full-text and chose the articles that were relevant to our systematic review.

Quality Assessment of the Studies

All papers that were included in the final shortlist were assessed using the following Quality Assessment Tools by all the authors. Observational studies were assessed using the Joanna Briggs Institute (JBI) Critical Appraisal Checklist, and the Newcastle-Ottawa Scale (NCOS) critical appraisal tool was used for cross-sectional studies, case-control studies, and cohort studies. We used the Scale for the Assessment of Narrative Review Articles (SANRA) critical appraisal tool for narrative review articles, and the Assessment of Multiple Systematic Review (AMSTAR) tool was used for systematic reviews. The authors assessed the papers using these tools and included good-quality papers in the systematic review.

Results

Study Identification

**Figure 1:**
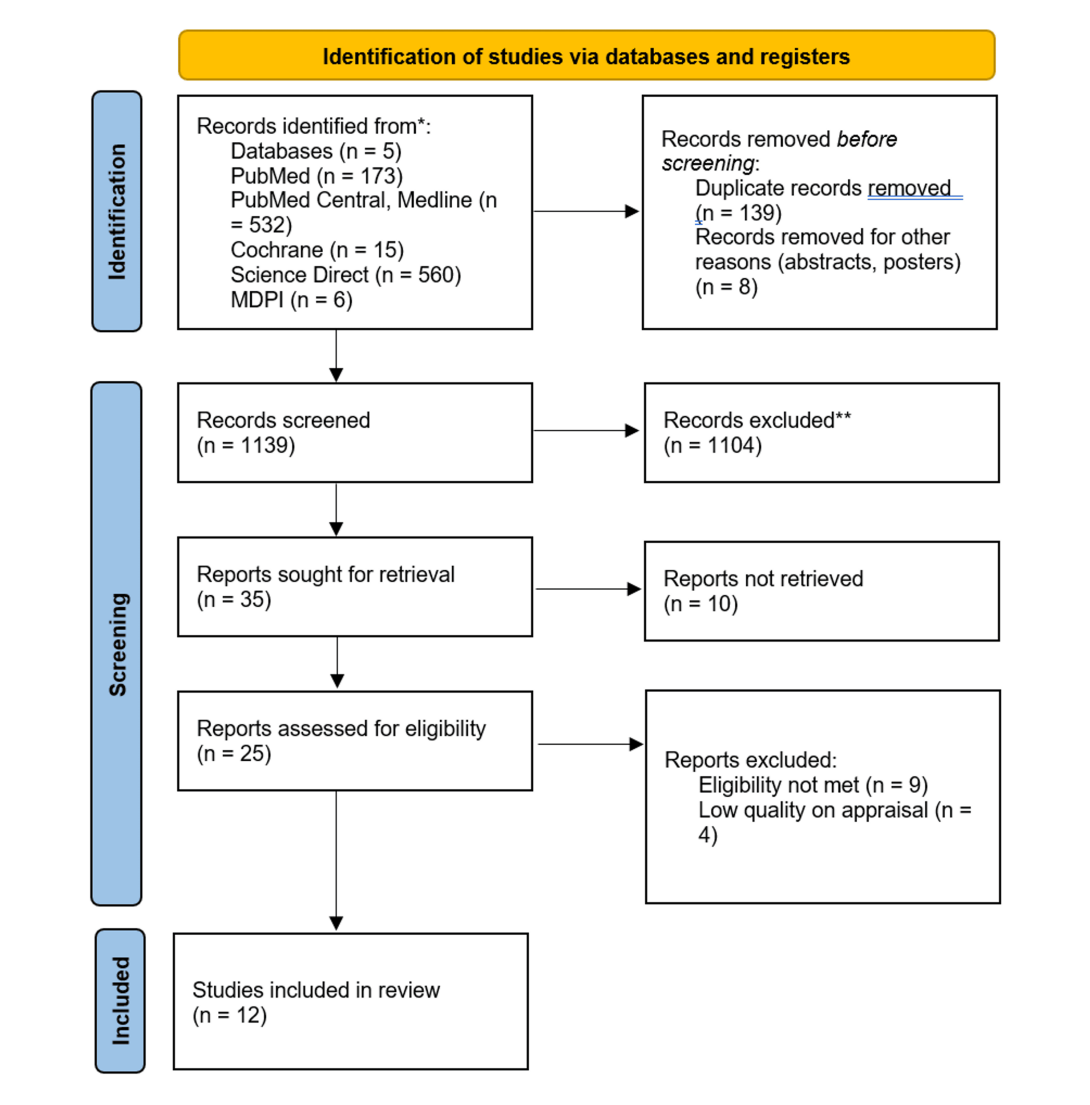
PRISMA chart depicting the selection of articles. *Consider, if feasible to do so, reporting the number of records identified from each database or register searched (rather than the total number across all databases/registers). **If automation tools were used, indicate how many records were excluded by a human and how many were excluded by automation tools. PRISMA: Preferred Reporting Items for Systematic Reviews and Meta-Analyses

We conducted a comprehensive search through five databases and identified 1,286 articles. After removing 139 duplicates, we screened the remaining papers by title and excluded those papers that were irrelevant. Next, we removed articles due to the unavailability of full-text, and we were then left with 35 papers. The full texts of these articles were retrieved, and following a detailed assessment, the number was narrowed to 25. A thorough assessment was conducted using quality assessment, and five papers did not meet the required target of 70%. In our systematic review, we included 12 articles that met the quality assessment standard. Figure 1 illustrates the detailed selection process in the form of a PRISMA flowchart.

Study Characteristics

We conducted a comprehensive review of 12 studies involving approximately 2,443 participants. Among these, six were cross-sectional, one was a case-control study, two were narrative reviews, and three were cohort studies. A detailed assessment of the included studies is presented in Tables 2-6.

**Table 2:** NCOS critical appraisal tool for cross-sectional studies. *Study meets the criterion for that specific item. **Study adequately controlled for two key confounders. NCOS: Newcastle-Ottawa Scale

Studies	Beling et al. (2021) [18]	Mayes et al. (2017) [21]	Beling et al. (2023) [17]	Gonzalez-Ruiz et al. (2023) [19]	Cațan et al. (2020) [22]	Herdea et al. (2020) [14]	Silva et al. (2017) [23]
Representativeness of the sample	*	*	*	*	*	*	*
Satisfactory sample size	*	No	*	*	No	No	No
Non-respondents	*	No	No	No	No	No	No
Validated risk-factor measurement	**	**	**	**	**	*	No
Comparable subjects in different outcome groups	*	*	**	*	**	**	*
Clear assessment of outcome	**	**	*	**	**	**	*
Clearly described statistical test	*	*	*	*	*	*	*
Total score (maximum stars)	9/10	7/10	8/10	8/10	8/10	7/10	4/10
Quality	Good	Good	Good	Good	Good	Good	Poor

**Table 3:** NCOS critical appraisal tool for assessment of case-control studies. *Study meets the criterion for that specific item. NCOS: Newcastle-Ottawa Scale

Studies	Balioglu et al. (2017) [2]	Danielewicz et al. (2023) [9]
Adequate case definition	*	*
Representativeness of the cases	*	*
Selection of controls	No	*
Definition of controls	*	No
Comparability	*	*
Ascertainment of exposure	*	*
Same method of ascertainment for cases and controls	*	*
Non-response rate	*	No
Total score (maximum stars)	7/9	6/9
Quality	Good	Poor

**Table 4:** NCOS critical appraisal tool for assessment of cohort studies. *Study meets the criterion for that specific item. **Study adequately controlled for two key confounders. NCOS: Newcastle-Ottawa Scale

Studies	Hampton et al. (2022) [16]	Alsiddiky et al. (2020) [8]	Sarkovich et al. (2024) [24]
Representativeness of the exposed cohort	*	*	*
Selection of the non-exposed cohort	No	No	*
Ascertainment of exposure	*	*	*
Demonstration that the outcome of interest was not present at the start of the study	*	No	No
Comparability	**	**	*
Assessment of outcome	*	*	*
Was the follow-up long enough?	*	*	*
Adequacy of follow-up of the cohort	*	*	*
Total score (maximum stars)	8/9	7/9	7/9
Quality	Good	Good	Good

**Table 5:** SANRA critical appraisal tool for assessment of narrative review articles. *Criterion partly fulfilled. **Criterion fully fulfilled. SANRA: Scale for the Assessment of Narrative Review Articles

Studies	Ng et al. (2018) [25]	Mobasseri (2025) [4]
Justification of the article’s importance	**	**
Statement of concrete aims or formulation of questions	**	**
Description of the literature search	No	No
Referencing	**	**
Scientific reasoning	**	**
Appropriate presentation of data	*	*
Total score (maximum stars)	9/12	9/12
Quality	Good	Good

**Table 6:** AMSTAR critical appraisal tool for assessment of systematic reviews. AMSTAR: Assessment of Multiple Systematic Review; PICO: Population, Intervention, Comparison, Outcome; RoB: Risk of Bias

Studies	Zhu et al. (2019) [26]	Kim and Lee (2023) [27]	Llopis-Ibor et al. (2023) [15]
Components of PICO	Yes	Yes	Yes
Established review methods	Partial yes	Partial yes	Partial yes
Selection of study designs explained	No	Yes	Yes
Comprehensive literature search strategy	Partial Yes	Yes	Yes
Study selection in the supplementary performed	Yes	Yes	Yes
Data extraction in duplicate	Yes	Yes	Yes
List of excluded studies	No	Yes	No
Describe the included studies in detail	Partial yes	Yes	Partial yes
RoB assessed	No	No	Yes
Sources of funding	No	No	No
Appropriate methods for statistical combination of results	Yes	Yes	No
Potential impact of RoB in individual studies	No	No	No
RoB in individual studies when discussing results	No	No	No
Satisfactory explanation for heterogeneity	Yes	Yes	Yes
Adequate investigation of publication bias	No	No	Yes
Report conflict of interest	Yes	Yes	Yes
Total score (maximum stars)	7.5/16	10.5/16	10/16
Quality	Poor	Poor	Poor

Outcomes Measured

We conducted a systematic review of studies involving patients diagnosed with AIS, in which the effect of VDD on surgical outcomes was assessed. We focused on the prevalence of VDD in the AIS population and on determining the impact of VDD on the patient during both pre-operative and post-operative periods. Comparisons between subjects who were supplemented with vitamin D pre-operatively and those who were not were made in some papers to assess the potential benefits of addressing deficiency before operative intervention. Moreover, we also examined the diverse clinical and biochemical factors associated with less-than-desirable surgical outcomes in AIS. Tables 7, 8 summarize the study characteristics of the articles included in our systematic review.

**Table 7:** Geographic characteristics of the included studies.

Studies	Design	Place	Age	Ratio Male:Female	Sample size	Time
Beling et al. (2021) [18]	Cross-sectional study	Boston, USA	10.6-25.3	32:144 (82%)	176	May 2015 to June 2017
Mayes et al. (2017) [21]	Cross-sectional study	Cincinnati, USA	10-17.2	72:145 (67%)	217	August 2012 to August 2014
Beling et al. (2023) [17]	Cross-sectional study	Cleveland, USA	10-18	15:72 (83%)	87	Sept 2016 to March 2020
Gonzalez-Ruiz et al. (2023) [19]	Cross-sectional study	Spain	10-16	8:45 (85%)	53	Oct 2021 to Feb 2022
Cațan et al. (2020) [22]	Cross-sectional study	Timisoara, Romania	13-16	100%	32	Jan 2019 to Sept 2019
Herdea et al. (2020) [14]	Cross-sectional study	Bucharest, Romania	8-16	25:76	101	June 2017 to July 2019
Balioglu et al. (2017) [2]	Case-control study	Istanbul, Turkey	10-22	334:284 (46%)	618	Jan 2012 to Oct 2014
Hampton et al. (2022) [16]	Cohort study	Sheffield, UK	13-18	24:177 (89%)	201	Jan 2014 to June 2019
Alsiddiky et al. (2020) [8]	Cohort study	Saudi Arabia	10-25	13:54 (80.6%)	67	Aug 2015 to Oct 2017
Sarkovich et al. (2024) [24]	Cohort study	New Orleans, USA	10-17	653:238 (73%)	891	Jan 2018 to Dec 2021
Ng et al. (2018) [25]	Narrative review	-	-	-	-	-
Mobasseri (2025) [4]	Narrative review	-	-	-	-	-

**Table 8:** Outcomes observed in the included studies. Y: yes/correlation between VDD and characteristic present; X: no/no reported correlation between VDD and characteristic; -: no comment in the article regarding the characteristic and connection to VDD; VDD: vitamin D deficiency

Studies	Prevalence	Back pain	Cobb angle	Recommended correction prior to surgery	Recommended screening and correction	Positive findings
Beling et al. (2021) [18]	High	X	Y	X	X	(1) High prevalence and (2) VDD is associated with increased Cobb angle.
Mayes et al. (2017) [21]	High	-	-	-	Y	(1) High prevalence
Beling et al. (2023) [17]	High	Y	-	Y	Y	(1) High prevalence
Gonzalez-Ruiz et al. (2023) [19]	-	-	Y (pre-menarchal group)	X	X	(1) High prevalence and (2) VDD is associated with increased Cobb angle.
Cațan et al. (2020) [22]	High	-	Y	-	-	(1) High prevalence and (2) VDD is associated with increased Cobb angle.
Herdea et al. (2020) [14]	High	-	Y	-	Y	(1) High prevalence and (2) VDD is associated with increased Cobb angle.
Hampton et al. (2022) [16]	High	Y	X	Y	Y	(1) High prevalence and (2) VDD is associated with back pain.
Alsiddiky et al. (2020) [8]	High	-	Y but not statistically significant	-	-	(1) High prevalence and (2) VDD is associated with increased Cobb angle.
Sarkovich et al. (2024) [24]	-	X	-	-	-	-
Balioglu et al. (2017) [2]	High	-	Y	-	Y	(1) High prevalence and (2) VDD is associated with increased Cobb angle.
Ng et al. (2018) [25]	-	-	-	-	-	-
Mobasseri (2025) [4]	-	-	-	-	-	-

Discussion

Prevalence of Vitamin D Insufficiency in AIS Patients

Several research studies show a high prevalence of VDD or insufficiency in adolescents with AIS [8,9,14,16,18]. Alsiddiky et al. found that 92.5% of the patients undergoing surgery for AIS had low vitamin D levels, with a mean serum level of 37.86±26 nmol/L (approximately 15.14 ng/mL) [8]. Another study published by Herdea et al. reported that nearly three-quarters (72.27%) of AIS patients had below normal vitamin D values, of which 40.59% were strictly deficient (<20 ng/mL) and 31.68% were insufficient (20-29 ng/mL), showing that even in those patients who were not strictly deficient, vitamin D status was suboptimal [14]. Additionally, a study held in the United Kingdom by Hampton et al., which included patients undergoing surgery for AIS, found that 74% of patients required supplementation with vitamin D, and only 5.5% had "normal" vitamin D levels (>75 nmol/L), indicating that adequate vitamin D status was the exception instead of the norm even in a pre-operative surgical population [16]. Beling et al. found that most AIS patients had hypovitaminosis D [18]. Similarly, Balioglu et al., Mayes et al., and Cațan et al. observed a similar pattern, where vitamin D levels were lower in patients diagnosed with AIS [2,21,22]. Collectively, these findings suggest that the majority of AIS patients, whether conservatively managed or planned for spinal surgery, have low vitamin D levels.

Correlation Between Back Pain and VDD in AIS Patients

The relationship between back pain scores and VDD remains debatable, with some studies showing a correlation between the two, while others do not. Beling et al. discovered that, two to 10 years following spinal fusion, patients with low vitamin D levels had poorer Function, Self-image, and Total SRS-30 scores [17]. A study by Hampton et al. reported a strong correlation between the intensity of back pain and the degree of VDD in AIS patients, suggesting that lower vitamin D levels would be related to the severity of pain. In this study, vitamin D replacement was required in 74% patients. Based on these findings, the authors recommend screening every patient with AIS for VDD and providing supplementation as needed, especially since it may improve pain outcomes post-operatively [16].

In contrast, however, another study found no notable relationship between the presence of back pain and VDD in diagnosed AIS patients [24]. This suggests that the correlation may not always be present. A study conducted by Beling et al., which included AIS patients who underwent spine fusion, found no correlation between vitamin D levels and pre-operative SRS scores across function, pain, self-image, mental health, and satisfaction domains. They also concluded that VDD showed no differences in pain during the first 72 h post-operative period. This study concluded that vitamin D status does not influence AIS patients' level of pain before or immediately after spine fusion surgery [18]. Therefore, the relation between pre-operative and post-operative pain and its correlation to VDD needs to be further investigated.

Correlation Between Cobb Angle and VDD in AIS Patients

A study conducted by Herdea et al. suggests a negative correlation between vitamin D levels and Cobb angle, suggesting that lower vitamin D concentrations may be associated with more severe spinal curvature in AIS patients [14]. They concluded that considering the correlation between calcium and vitamin D, as well as the negative correlation with Cobb angle, indicates that patients with AIS should be routinely screened for VDD [14]. A case-control study conducted by Balioglu et al. found a negative correlation between Cobb angle and VDD (p<0.026, r = -0.147) [2]. Another study by González-Ruiz et al. also found a strong negative correlation (r = -0.92) between serum vitamin D levels and the main Cobb angle. However, this association was only evident in premenarcheal females (n=7) [19].

Beling et al. noted a weak negative correlation, indicating that AIS patients with lower pre-operative vitamin D levels often presented with larger major curve magnitudes initially. Interestingly, this study also found that vitamin D-deficient patients were less frequently prescribed bracing and, when bracing was used, the treatment duration was shorter compared to those with adequate vitamin D status [18]. Cațan et al. conducted a cross-sectional study which observed that AIS patients had lower vitamin D levels when compared to the control group and henceforth a low bone mineral density (especially in individuals aged 13 to 17 years old). They also observed that Cobb angle correlated significantly with DEXA Z-score: Cobb angle and Z-score were inversely proportional [22].

On the other hand, two studies included in our analysis failed to demonstrate a significant correlation between vitamin D levels and Cobb angles [8,16]. Alsiddiky et al. found that although vitamin D insufficiency was associated with higher Cobb angles, this association did not reach statistical significance, as indicated by the calculated p-value. They found that the mean Cobb angle was 57.62±20.5°, with a maximum of 122° [8]. These findings suggest that although it is not universally proven that there is major significant statistical correlation between vitamin D insufficiency/deficiency and Cobb angle, most studies show that low levels of vitamin D do lead to worse Cobb angles, and the results could vary depending on study design, sample characteristics, or the method used.

Therapeutic and Surgical Implications

The effect of vitamin D on post-operative outcomes in the AIS population remains unknown. Beling et al. postulated that weaker spine fusion and pedicle screw purchase may be seen in AIS patients with vitamin D insufficiency. In theory, this might put extra strain on implants and jeopardize fixation, aggravating the bone-muscle interface [18].

According to Hampton et al., patients with AIS must be routinely screened for vitamin D levels and optimized with supplementation, especially pre-operatively. This is suggested due to the high prevalence of VDD among patients, as well as the possible effect on bone health and back pain that could affect the clinical outcomes. This study accounted for variables like age, sun exposure, and seasonal variation, which could potentially impact VDD [16]. Vitamin D plays an important role in muscle strength, immune response, and the scarring process, and correcting this deficiency before surgery is a relatively simple and cost-effective way to improve overall patient outcomes.

Regarding the direct impact of vitamin D status on operative results and post-operative complications, however, the current evidence base remains inconclusive. Beling et al. investigated pre-operative, intra-operative, and early post-operative factors and were not able to identify any significant differences in AIS patients with normal, insufficient, and deficient vitamin D levels. This study considered potential confounders, including age, race, ethnicity, income, season, and second-hand smoke exposure, using multivariable modelling. The authors noted that intra-operatively, the number of spinal levels fused, surgical time, and estimated intra-operative blood loss were equivalent between groups. Post-operatively, there were no differences in the degree of major curve correction achieved, rates of morphine use, or hospital stay time during the early post-operative period. These findings suggest that vitamin D status may not play a significant role in patients' pre- or early post-operative pain, or peri-operative outcomes [18].

More recent research by Beling et al., however, suggests a possible longer-term impact on patient-reported outcomes. This study noted that patients with VDD presented for AIS surgery at a younger age (2.5 years younger than insufficient patients and three years younger than patients with normal vitamin D levels). Beling et al. also reported that vitamin-D-deficient AIS patients had lower SRS-30 Function score (p=0.002), self-image score, and total score compared to their vitamin D-deficient counterparts 2-10 years after spinal surgery. The mean function score in vitamin D-deficient patients was 3.3 (SD±0.41), similar to inadequate patients, significantly lower than that of sufficient patients, the mean score of which was 3.6 (SD±0.42) (p=0.009). The mean self-image domain score was 3.7 (SD±0.61) in vitamin D-deficient patients, compared to 3.9 (SD±0.49) in patients with inadequate vitamin D levels. This was less than 4.2 (SD±0.49) in patients with sufficient levels (p=0.004). Mean total SRS scores for vitamin D-deficient patients were 3.7 (SD±0.41), no different from those of patients with insufficient levels (mean 3.7; SD±0.46), and significantly lower than for patients with sufficient levels (mean 4.0; SD±0.41) (p=0.02). The association persisted even after adjusting for age, body mass index, race, ethnicity, and Fitzpatrick's score. This study also considered whether the lower Function, Self Image, and Total Scores could be attributed to demographic and socioeconomic factors and found that the correlation persisted [17]. This suggests that, although vitamin D levels may not have an impact pre-operatively, intra-operatively, or during initial recovery post-operatively, deficiency can have a detrimental effect on long-term functional recovery and overall quality of life following spinal fusion surgery, and hence, affects long-term patient outcomes [17]. Taken together, these findings are indicative of the potential importance of pre-operative optimization of vitamin D levels.

Results From the Narrative Reviews Included in Our Study

We included two narrative reviews in this study [4,25]. Ng et al. reviewed existing literature to explore the association between vitamin D status and the pathogenesis of AIS. The study showed that vitamin D had a positive correlation with bone mineral density and was negatively correlated with the Cobb angle. Furthermore, vitamin D has been demonstrated to interact with other hormones, such as estrogen, melatonin, and leptin, that affect bone metabolism and AIS etiology. It concluded that VDD and insufficiency are prevalent in adolescents with AIS [25].

Mobasseri suggests that pre-operative VDD can lead to poor post-operative outcomes in AIS patients and identifies other factors that can exacerbate VDD post-operatively. ICU admission following AIS surgery can lead to exacerbation of VDD due to surgical stress, inflammatory mediator release, opioid use, hormone changes, or electrolyte and hydration imbalances. Post-operative pain can also lead to reduced dietary intake, mobilization, and physical activity. Moreover, analgesics can interfere with vitamin D metabolism, causing a drop in vitamin D levels. Morphine can also cause nausea and vomiting, which leads to decreased oral intake. The surgical stress response leads to increased cortisol levels, which can increase calcium excretion, degrade vitamin D, and interfere with its absorption and function. Hypocalcemia can further cause tissues to utilize the existing vitamin D in the body, worsening vitamin D levels. Post-operative complications like infections or neurological deficits, which can prolong hospital stay and limit physical activity, also worsen VDD. Bracing may also contribute to VDD by reducing sun exposure due to overdressing or outdoor avoidance caused by discomfort [4].

Effect of VDD in Patients Undergoing Spinal Fusion

We also wanted to discuss the implications of VDD in patients undergoing spinal fusion surgery, which is the most common surgery performed in AIS patients. Vitamin D plays an important role in bone mineralization, bone remodeling, and calcium homeostasis [28]. Because of this, VDD affects bone hemostasis, causing low bone mineral density, which has been further linked to pseudoarthrosis and spinal instrumentation failure. VDD also increases the risk of fractures and has been linked to poor patient outcomes in patients undergoing spinal fusion [28].

Kerezoudis et al. included five clinical studies (two prospective-controlled, two retrospective studies, and one case series) in a systematic review (based on PRISMA), and it included 264 patients who were undergoing lumbar fusion, to determine the relationship between vitamin D status and outcomes after spinal arthrodesis. Of these, 122 patients (45%) were vitamin D deficient or insufficient [28]. According to Ravindra et al., patients with VDD experienced a significantly increased fusion time (median 12 vs. six months, p<0.001) and more than three times greater risk of non-union in deficient patients (OR=3.449, 95% CI: 1.029-11.561). This correlation persisted even after adjusting for potential confounders [29]. Kim et al. reported that VDD was associated with a lower quality of life (EQ-5D score of 0.411 vs. 0.657) and higher post-operative disability scores (Oswestry Disability Index 43.1 vs. 19.9). Most significantly, supplementation seemed to reverse these adverse effects in deficient patients [30]. Patients who took supplements following transforaminal lumbar interbody fusion had a noticeably higher rate of fusion at 95.24%, compared to 65.22% with a placebo (p=0.02), according to Xu et al. [31]. In a different series, Waikakul reported better pain and function, with mean Japanese Orthopaedic Association scores ranging from 7.6 to 1.1 (p<0.001) and visual analog scores ranging from 7.7 to 4.2 [32]. As observed across the included studies, deficiency was consistently linked with poorer fusion outcomes and worse patient-reported measures [28]. They recommended routine assessment and correction of VDD in patients undergoing spinal fusion.

Limitations

This systematic review has a few limitations. We have only analyzed the patient qualitatively. A quantitative analysis, such as a meta-analysis of this study, has not yet been conducted, which would provide deeper insight into the relationship between VDD and surgical AIS patients. Moreover, although the relationship between VDD and its role in the development of AIS has been increasingly suggested by the current research, there are not many papers published regarding the implications of VDD pre-operatively and post-operatively in surgical AIS patients.

We suggest conducting further research and statistical analysis on the effect of VDD on surgical AIS patients. Furthermore, additional research should address key confounding variables, such as age, BMI, ethnicity, seasonality, calcium intake, and sun exposure. This can be achieved by collecting standardized information on these factors and adjusting for them in multivariable analysis, or by using stratification, matching, or restriction as appropriate.

## Conclusions

This systematic review investigated the interaction between VDD and the outcomes of spinal fusion surgery in AIS patients. Current evidence is insufficient to demonstrate that vitamin D supplementation pre-operatively will lead to better surgical outcomes in AIS patients. However, previous research suggests that vitamin D supplementation before spinal fusion is necessary, but this has not been adequately examined in AIS patients. Further multicenter studies are required to conclude the role of vitamin D in AIS etiopathogenesis, its impact on curve progression, and its long-term effects on surgical outcomes and patient-reported quality of life measures. Overall, it can be concluded that while VDD is prevalent in AIS patients and there are some studies suggesting better spinal fusion outcomes, the current evidence is insufficient to support routine screening or supplementation as a standard recommendation.
